# DOCK2 confers immunity and intestinal colonization resistance to *Citrobacter rodentium* infection

**DOI:** 10.1038/srep27814

**Published:** 2016-06-13

**Authors:** Zhiping Liu, Si Ming Man, Qifan Zhu, Peter Vogel, Sharon Frase, Yoshinori Fukui, Thirumala-Devi Kanneganti

**Affiliations:** 1 Department of Immunology, St. Jude Children’s Research Hospital, Memphis, TN, 38105, USA; 2 Integrated Biomedical Sciences Program, University of Tennessee Health Science Center, Memphis, Tennessee 38163, USA; 3 Animal Resources Center and the Veterinary Pathology Core, St. Jude Children’s Research Hospital, Memphis, TN, 38105, USA; 4 Cell and Tissue Imaging Center, St. Jude Children’s Research Hospital, Memphis, TN, 38105, USA; 5 Division of Immunogenetics, Department of Immunobiology and Neuroscience, Medical Institute of Bioregulation, Kyushu University, Fukuoka 812-8582, Japan

## Abstract

Food poisoning is one of the leading causes of morbidity and mortality in the world. *Citrobacter rodentium* is an enteric pathogen which attaches itself to enterocytes and induces attachment and effacing (A/E) lesions. The ability of the bacterium to cause infection requires subversion of the host actin cytoskeleton. Rac-dependent actin polymerization is activated by a guanine nucleotide exchange factor known as Dedicator of cytokinesis 2 (DOCK2). However, the role of DOCK2 in infectious disease is largely unexplored. Here, we found that mice lacking DOCK2 were susceptible to *C. rodentium* infection. These mice harbored increased levels of *C. rodentium* bacteria, showed more pronounced weight loss and inflammation-associated pathology, and were prone to bacterial dissemination to the systemic organs compared with wild-type mice. We found that mice lacking DOCK2 were more susceptible to *C. rodentium* attachment to intestinal epithelial cells. Therefore, our results underscored an important role of DOCK2 for gastrointestinal immunity to *C. rodentium* infection.

The human enteric pathogens enteropathogenic *Escherichia coli* (EPEC) and enterohemorrhagic *E. coli* (EHEC) are major causes of food poisoning^1^. Infection by EPEC is associated with childhood mortality in developing countries, whereas infection by EHEC causes hemolytic uremic syndrome^2^,^3^. Attachment to intestinal epithelial cells by EPEC and EHEC induces distinctive pedestal-like structures on the host cell surface known as attaching and effacing (A/E) lesions. A related A/E-associated pathogen *Citrobacter rodentium* is used extensively to study the host-microbe relationship in mouse models^4^,^5^.

Mice infected with *C. rodentium* are susceptible to weight loss and develop soft stool and epithelial crypt hyperplasia^6^,^7^. Like EPEC and EHEC, the genome of *C. rodentium* contains a pathogenicity island known as the locus of enterocyte effacement (LEE)^8^. The LEE contains genes encoding a type III secretion system, a molecular syringe used by bacteria to inject virulence-associated proteins into the host cell in order to subvert its functions and to enhance the development of disease. The LEE-encoded proteins translocating intimin receptor (Tir) and the bacterial outer membrane adhesin intimin have roles in bacterial virulence and the formation of A/E lesions^9^. Tir is translocated into the host cell by the type III secretion system to serve as a receptor for intimin^9^,^10^,^11^,^12^. These proteins are necessary for inducing cytoskeletal rearrangements and actin-rich pedestal formation^10^,^11^.

Actin polymerization is an important innate immune mechanism which controls bacterial infection^13^. Rac-dependent actin polymerization is activated by the guanine nucleotide exchange factor Dedicator of cytokinesis 2 (DOCK2), a mammalian homolog of CED-5 from *Caenorhabditis elegans* and myoblast city (MBC) from *Drosophila melanogaster*^14^,^15^. The SH3 domain of DOCK2 associates with the C-terminal sequence of engulfment and cell motility (ELMO1)^15^,^16^,^17^. This interaction relieves autoinhibition of DOCK2, which is then allowed to fully activate Rac1. The importance of DOCK2 in the immune system is indicated by its expression in monocytes, macrophages, lymphocytes and other hematopoietic cells^18^,^19^,^20^. DOCK2 functions synergistically with DOCK5 to induce PMA-induced Rac activation, ROS production and formation of neutrophil extracellular traps in mouse neutrophils^21^. Importantly, dendritic cells defective in DOCK2 exhibit impaired endocytosis of soluble antigens and phagocytosis of insoluble antigens and larger particles^22^. In addition, DOCK2 contributes to T and B cell migration^14^,^23^. These findings highlight important roles of DOCK2 in the immune system. However, the function of DOCK2 in immunity to infectious diseases remains unknown.

Here, we showed that mice lacking DOCK2 were more susceptible to enteric *C. rodentium* infection. Mice lacking DOCK2 were prone to bacterial dissemination to the systemic organs, had an impaired ability to recruit immune cells and had a reduced capacity to prevent rapid bacterial attachment to the intestinal epithelium compared with wild-type mice. These findings identified DOCK2 as a critical regulator of gastrointestinal immunity to the enteric pathogen *C. rodentium*.

## Results

### DOCK2 provides host resistance to *C. rodentium* infection

**Figure 1:**
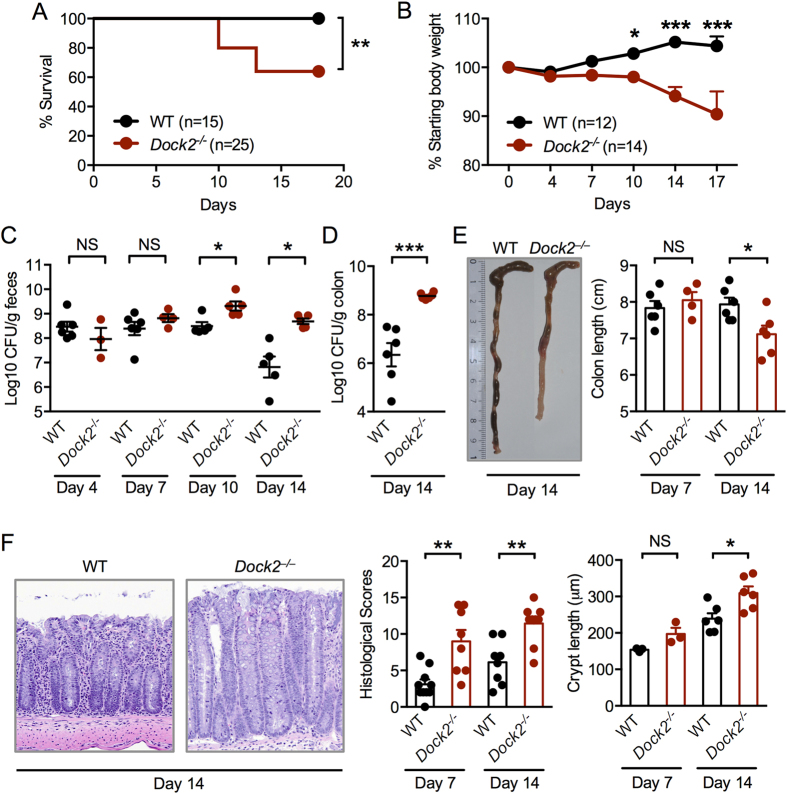
DOCK2 is required for host protection against *C. rodentium* infection. (**A**,**B**) Survival and body weight change of WT and *Dock2*^−/−^ mice orally infected with 1 × 10^10^ CFU of *C. rodentium*. (**C**,**D**) *C. rodentium* CFU in fecal and colon samples. (**E**) Lengths of the colons on Days 7 and 14. (**F**) H&E staining of colon tissues and quantification of crypt length and intestinal damage. Each symbol represents an individual mouse. Data are representative of three independent experiments (mean and SEM). (**A**) Log-rank test. (**B**) Two-way ANOVA. (**C**–**F**) Two-tailed *t*-test. *P < 0.05; **P < 0.01; ***P < 0.001; ****P < 0.0001; NS, not statistically significant.

We infected wild-type (WT) and *Dock2*^−/−^ mice via oral gavage with 1 × 10^10^ CFU of *C. rodentium* and monitored their survival for 18 days. All WT mice controlled and survived the infection (Fig. 1A), consistent with the phenotype of self-limiting colitis induced by *C. rodentium*^6^. Compared with WT mice, *Dock2*^−/−^ mice were significantly more susceptible, with 39% of the *Dock2*^−/−^ mice succumbing to the infection by day 13 (*P* < 0.01; Fig. 1A). *Dock2*^−/−^ mice lost body weight, especially 10 days after infection (Fig. 1B). Compared with infected WT mice, we found a significantly increased burden of *C. rodentium* bacteria in the stool of infected *Dock2*^−/−^ mice on days 10 and 14, but not on days 4 and 7 (Fig. 1C). A significantly higher bacterial number was observed in the colon of *Dock2*^−/−^ mice on day 14 compared with WT mice (Fig. 1D). Shortening of the cecum and colon – a hallmark of colitis – was more pronounced in infected *Dock2*^−/−^ mice on day 14 compared with infected WT mice (Fig. 1E).

The increased susceptibility of *Dock2*^−/−^ mice to *C. rodentium* infection was validated by histological analysis. Increased crypt lengths and levels of transmissible murine crypt hyperplasia owing to thickening of the mucosa were found in infected *Dock2*^−/−^ mice on day 14 (Fig. 1F)^7^. Histological analyses also revealed that *Dock2*^−/−^ mice developed more severe lesions than WT mice on both days 7 and 14 after *C. rodentium* infection (Fig. 1F). These results collectively suggested that DOCK2 contributed to the host protection against *C. rodentium* infection.

### DOCK2 mediates resistance to *C. rodentium* dissemination but is dispensable for the production of cytokines or anti-microbial peptides

**Figure 2:**
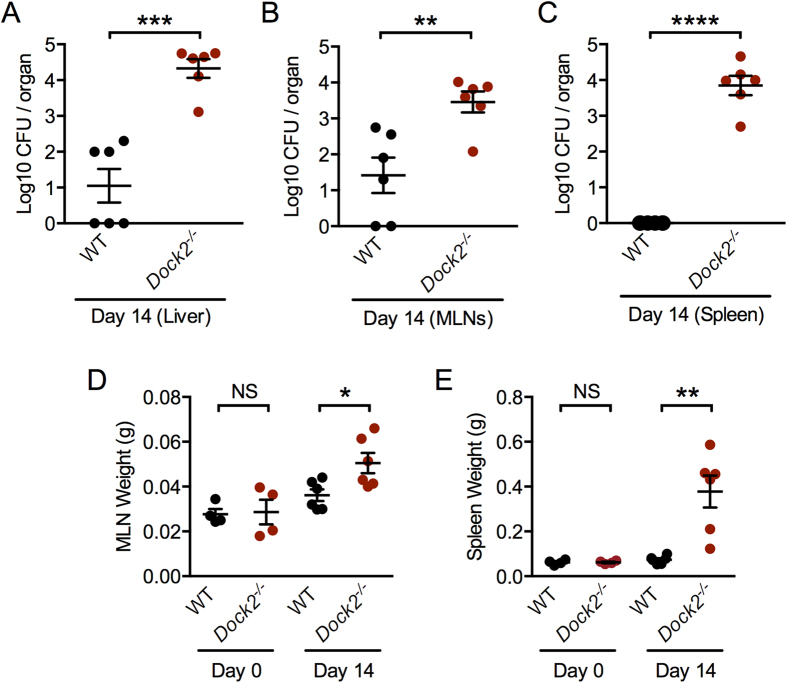
DOCK2 mediates resistance to *Citrobacter* dissemination into systemic organs. (**A**–**C**) WT and *Dock2*^−/−^ mice were orally infected with 1 × 10^10^ CFU of *C. rodentium*. The bacterial load was determined in the liver, MLNs and spleen 14 days post-infection. (**D**,**E**) The weight of the MLNs and spleen on Days 0 and 14 post-infection. Each symbol represents an individual mouse. Data are representative of two independent experiments (mean and SEM). Two-tailed t-test. *P < 0.05; **P < 0.01; ***P < 0.001; ****P < 0.0001; NS, not statistically significant.

A consequence of certain enteric bacterial infection is a breach of the intestinal barrier, causing bacterial dissemination from the gut to the systemic organs of a host. The increased fecal and colon *C. rodentium* burden in *Dock2*^−/−^ mice led us to hypothesize that bacteria are readily disseminated to the systemic organs of these mice, which could be responsible for the increased susceptibility and rate of mortality (Fig. 1). We infected WT and *Dock2*^−/−^ mice via oral gavage with 1 × 10^10^ CFU *C. rodentium* per mouse, harvested the spleen, liver and mesenteric lymph nodes (MLNs) 14 days post-infection and analyzed the presence of viable bacteria. We observed significantly more bacteria in the liver and MLNs of infected *Dock2*^−/−^ mice compared with infected WT mice (Fig. 2A,B). No viable bacteria was detected in the spleen of WT mice, whereas 10^4^ CFU of *C. rodentium* were found in the spleen of *Dock2*^−/−^ mice on day 14 (Fig. 2C). Accordingly, infected *Dock2*^−/−^ mice had enlarged spleen and MLNs compared with infected WT mice (Fig. 2D,E).

**Figure 3:**
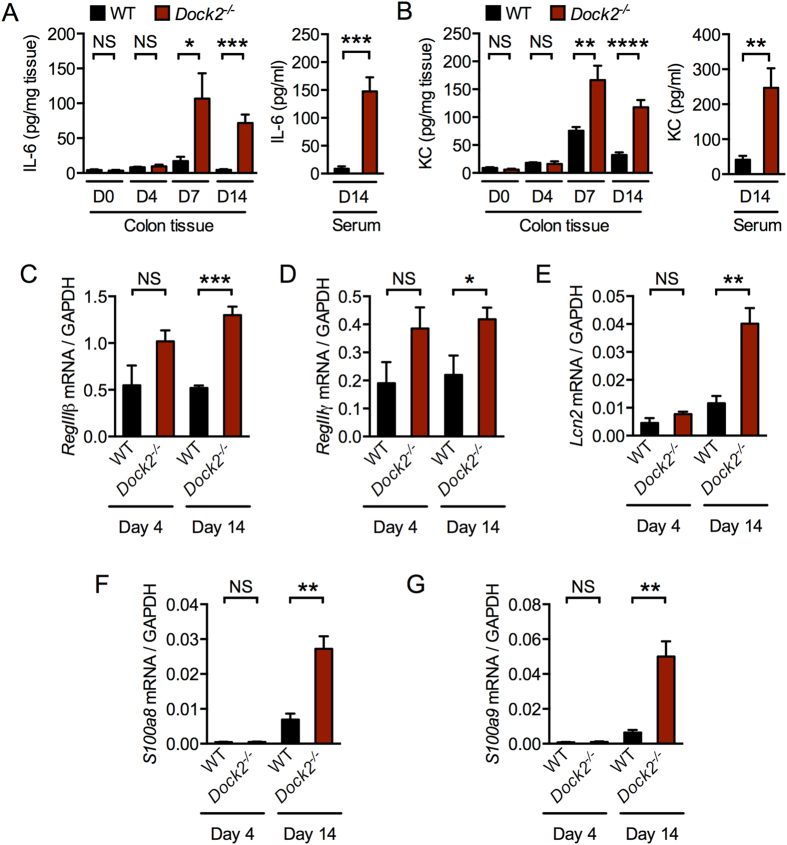
Production of pro-inflammatory cytokines and anti-microbial peptides in WT and *Dock2*^−/−^ mice. (**A**,**B**) The levels of IL-6 (**A**) and KC (**B**) proteins in the colon and serum in uninfected WT and *Dock2*^−/−^ mice or mice that had been infected with *C. rodentium*. (**C**–**G**) Real time qRT-PCR analysis of the expression of the gene encoding RegIIIβ (**C**), RegIIIγ (**D**), LCN2 (**E**), S100A8 (**F**), and S100A9 (**G**) in colon tissues of WT and *Dock2*^−/−^ mice infected with *C. rodentium*. Data are representative of two independent experiments (mean and SEM). Two-tailed *t*-test. *P < 0.05; **P < 0.01; ***P < 0.001; ****P < 0.0001; NS, not statistically significant.

The production of protective cytokines and anti-microbial peptides are hallmarks of immune responses being mounted towards the infection. We found significantly elevated levels of the pro-inflammatory cytokines, IL-6 and KC (also known as CXCL1) in the colon tissues of infected *Dock2*^−/−^ mice on days 7 and 14 compared with infected WT mice (Fig. 3A,B). We confirmed these results and found elevated circulating IL-6 and KC in the sera of *Dock2*^−/−^ mice (Fig. 3A,B). No difference in the levels of these cytokines between uninfected WT and *Dock2*^−/−^ mice and between infected WT and *Dock2*^−/−^ mice 4 days post-infection was observed (Fig. 3A,B). Moreover, we measured the levels of additional cytokines that have been shown to orchestrate immunological functions against *C. rodentium* infection, including IL-17, IFN-γ and TNF^7^. We found similar levels of IL-17 and IFN-γ in the colon tissues of infected WT mice and *Dock2*^−/−^ mice and elevated levels of TNF in the colon tissues of infected *Dock2*^−/−^ mice compared with WT mice 14 days post-infection (Supplementary Fig. S1A).

IL-22 and IL23 have emerged as key players in the host protection against *C. rodentium* infection^24^,^25^,^26^,^27^. Of particular importance is that IL-23 drives IL-22-mediated production of antimicrobial peptides within the Reg family, RegIIIβ and RegIIIγ, which critically provides early defense against *C. rodentium* infection^26^. We measured the expression of the genes encoding IL-22, IL-23p19, and the anti-microbial peptides RegIIIβ and RegIIIγ in the colon tissues of WT and *Dock2*^−/−^ mice. We found similar expression levels of the gene encoding IL-23p19 in the colon tissues between WT and *Dock2*^−/−^ mice on both days 4 and 14 (Supplementary Fig. S1B). The expression of the genes encoding IL-22, RegIIIβ and RegIIIγ were even elevated in infected *Dock2*^−/−^ mice on both days 4 and 14 (Supplementary Fig. S1C; Fig. 3C,D).

*C. rodentium* infection also induces production of the anti-microbial peptides LCN2, S100A8 and S100A9^28^. However, we found similar levels of these mediators in the colon tissues of WT and *Dock2*^−/−^ mice 4 days after infection and increased expression of these mediators after 14 days of infection (Fig. 3E–G). Overall, DOCK2 deficiency did not impair the production of protective pro-inflammatory cytokines and anti-microbial peptides at earlier time points during the infection. The levels of certain pro-inflammatory cytokines and anti-microbial peptides were elevated in infected *Dock2*^−/−^ mice predominately at later time points, which could be a consequence of the increased number of bacteria in their colon tissues. Taken together, these results suggested that the susceptibility of *Dock2*^−/−^ mice to *C. rodentium* infection was largely not owing to the inability to the host to produce pro-inflammatory cytokines and antimicrobial peptides.

### DOCK2 is required for infiltration of additional immune cells at later stages of infection

**Figure 4:**
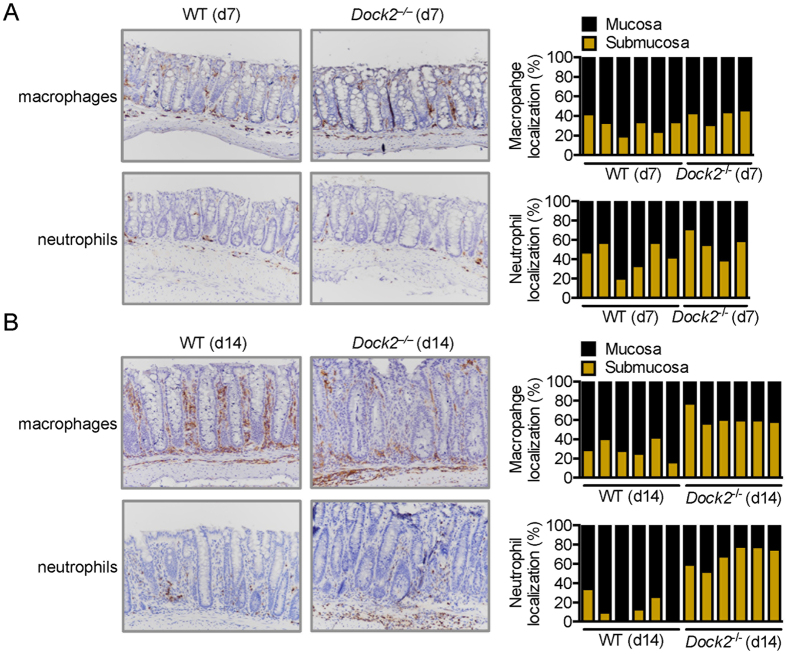
DOCK2 is required for infiltration of immune cells at late stages of infection with *C. rodentium*. (**A**,**B**) WT and *Dock2*^−/−^ mice were orally infected with 1 × 10^10^ CFU of *C. rodentium*. Immunohistochemistry staining of macrophages and neutrophils from colon tissue sections on Day 7 (**A**) and 14 (**B**) post-infection. Relative percentages of macrophages or neutrophils in the mucosa verses their relative percentage in the submucosa per field in WT mice (Day 7, n = 6; Day 14, n = 6) and *Dock2*^−/−^ mice (Day 7, n = 4; Day 14, n = 6) were determined. At least five different fields for each mouse were quantified. Each bar presents an individual mouse.

To investigate the role of DOCK2 in mucosal immunity against *C. rodentium* infection, we used immunohistochemistry techniques to localize macrophages and neutrophils in colon tissues of WT and *Dock2*^−/−^ mice infected with *C. rodentium*. Both macrophages and neutrophils were found in the colon of WT and *Dock2*^−/−^ mice 7 days after infection (Fig. 4A). We counted the number of macrophages and neutrophils localized to the mucosa versus the number localized to the submucosa to determine the relative distribution of these cells at these sites. We observed that the relative distribution of both immune cell types in the mucosa and submucosa was similar in WT and *Dock2*^−/−^ mice 7 days post-infection (Fig. 4A). However, we found a reduced proportion of the total macrophages and neutrophils infiltrating the mucosa in infected *Dock2*^−/−^ mice compared with infected WT mice 14 days after infection (Fig. 4B). Abundant numbers of macrophages and neutrophils were found in the submucosa of *Dock2*^−/−^ mice which failed to infiltrate the lamina propria (Fig. 4B). These data suggested that DOCK2 mediated infiltration of macrophages and neutrophils into the mucosa at later stages of *C. rodentium* infection.

### DOCK2 impairs bacterial attachment and formation of A/E lesions

The differential ability of macrophages and neutrophils to infiltrate the mucosa in WT and *Dock2*^−/−^ mice at the later stages of infection could be influenced, in part, by earlier events that establish the infection. *C. rodentium* employs an attaching and effacing mechanism to attach to enterocytes. This host-pathogen interaction is characterized by effacement of the brush border microvilli and the formation of pedestal-like structures on the host enterocyte^7^. We hypothesized that DOCK2 is able to prevent bacterial attachment to enterocytes at the earlier stages of *C. rodentium* infection, which is responsible for the differential production cytokines and antimicrobial peptides, infiltration of immune cells and bacterial dissemination.

**Figure 5:**
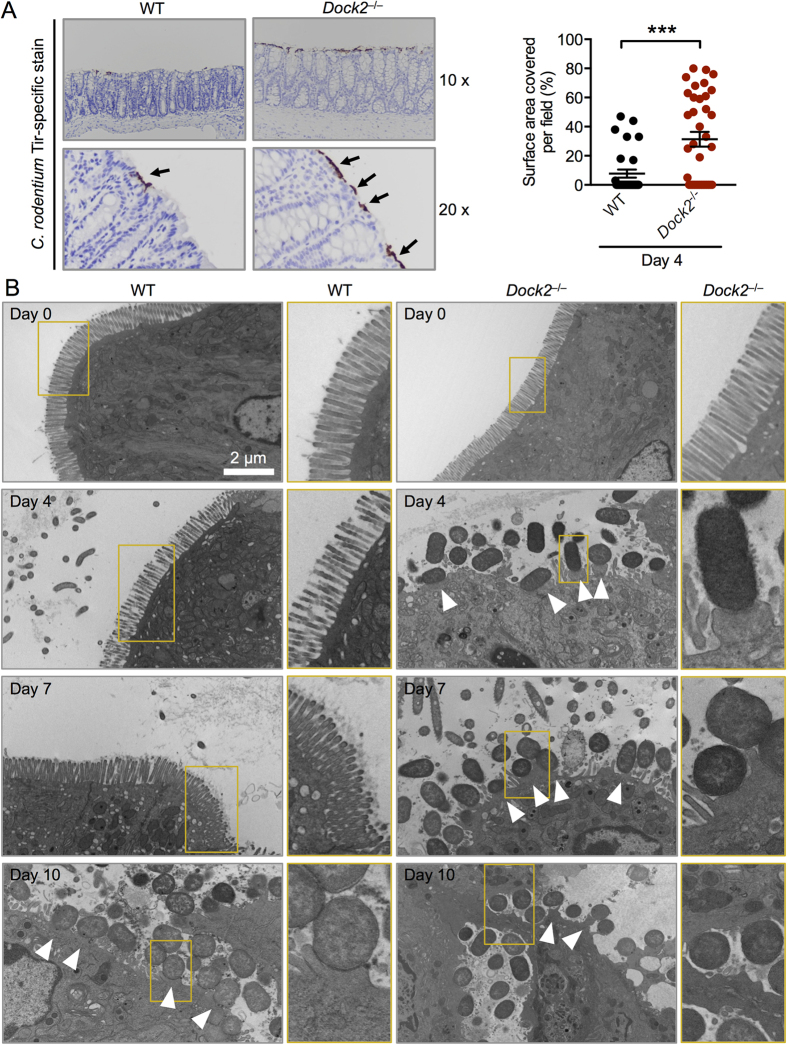
DOCK2 impairs bacterial attachment and formation of A/E lesions by *C. rodentium.* (**A**) WT and *Dock2*^−/−^ mice were orally infected with 1 × 10^10^ CFU of *C. rodentium*. Intestinal tissue sections were stained with anti-*C. rodentium* Tir antibody. Percentages of the area of the distal colon surface positive for Tir staining per field in infected WT mice (n = 5) and infected *Dock2*^−/−^ mice (n = 6). Six different fields for each mouse were quantified. (**B**) Transmission electron microscopy images of the colonic intestinal epithelium of WT and *Dock2*^−/−^ mice on Day 0 (uninfected), Day 4, Day 7 and Day 10 post-infection with *C. rodentium*. Arrowheads indicate bacterial attachment and formation of A/E lesions. Data are representative of two independent experiments (mean and SEM). Two-tailed *t*-test. ***P < 0.001.

To visualize attachment of bacteria to the intestinal epithelium, we performed immunostaining of the *C. rodentium* virulence factor Tir on colon sections from WT and *Dock2*^−/−^ mice. Tir is expressed during infection and is translocated into host cells via the Type III secretion system to mediate actin rearrangements and pedestal formation^10^. We observed an increased frequency of Tir staining covering the intestinal epithelial surface of the distal colon in *Dock2*^−/−^ mice compared with WT mice 4 days post-infection (Fig. 5A). To investigate whether *C. rodentium* is directly attaching to the intestinal epithelial cells and whether DOCK2 might be influencing this process, we performed transmission electron microscopy to visualize the bacteria and the presence of associated A/E lesions. We found an intact intestinal epithelium in both uninfected WT and *Dock2*^−/−^ mice (day 0, Fig. 5B). Remarkably, we frequently identified attachment of *C. rodentium* to microvilli and induction of pedestal-like structures on enterocytes along the colon of *Dock2*^−/−^ mice 4 and 7 days after infection (Fig. 5B). In contrast, we did not readily observe these events in WT mice on days 4 and 7. However, we frequently observed attachment of *C. rodentium* and destruction of microvilli in both WT and *Dock2*^−/−^ mice on day 10 (Fig. 5B). *C. rodentium* bacteria were also found throughout the mucosa and in the submucosa of *Dock2*^−/−^ mice. The increased ability of *C. rodentium* to attach to enterocytes of *Dock2*^−/−^ mice was not due to differential expression of genes encoding mucin (Supplementary Fig. S2). Indeed, the expression of genes encoding MUC1, MUC2, MUC3 and MUC4 was similar in the colon tissues of WT and *Dock2*^−/−^ mice (Supplementary Fig. S2). These results showed that *C. rodentium* attachment to enterocytes and microvilli destruction occurred earlier in mice lacking DOCK2 compared with WT mice.

## Discussion

Genetic studies revealed that mutations in the gene encoding DOCK2 are associated with colorectal cancer and esophageal adenocarcinoma^29^,^30^, raising the possibility that DOCK2 might play an important role in maintaining homeostasis of mucosal surfaces. However, the physiological function of DOCK2 in infectious diseases has remained undefined. A recent study identified in five children biallelic mutations in the gene encoding DOCK2 which largely impair expression of the DOCK2 protein^31^. These children all suffered multiple immunological dysfunctions and were susceptible to early invasive bacterial and viral infection^31^. Additional clues for a role of DOCK2 in immunity to infectious disease were highlighted in a study showing that a virulence factor known as Nef encoded by HIV-1 associates with DOCK2 to inhibit chemotaxis of T cells^32^. This finding suggests that HIV-1 has evolved strategies to specifically prevent DOCK2 functions to overcome the immune system.

Our study identified an important role for DOCK2 in the host defense against the pathogenic bacterium *C. rodentium* in mice. Infiltration of immune cells to the site of infection is crucial for host defense against pathogens and for mediating their clearance^7^. We found that DOCK2 is required for recruitment of macrophages and neutrophils to the mucosa where attachment and colonization of *C. rodentium* occurs. The impaired ability of macrophages and neutrophils to migrate to the mucosa may explain the defective bacterial clearance at the later stages of *C. rodentium* infection in mice lacking DOCK2. The gene encoding *Dock2* is predominately expressed in hematopoietic cells^14^,^18^,^19^,^20^. Therefore, it is likely that *Dock2*-deficient macrophages and neutrophils have an intrinsic defect in their ability to infiltrate the mucosa during *C. rodentium* infection. Given the established role for DOCK2 in orchestrating actin reorganization, it is possible that macrophages lacking DOCK2 may have an impaired ability to induce bacterial uptake even if they encounter the pathogen. Actin polymerization and cell-autonomous immunity are inextricably linked, which allows immune cells such as macrophages to phagocytose and control the number of bacteria per cell^13^,^33^. Indeed, we previously demonstrated that dendritic cells defective in DOCK2 exhibit impaired endocytosis of soluble antigens or phagocytosis of insoluble antigens and larger particles^22^, suggesting that DOCK2 is necessary for the engulfment process.

To identify earlier events leading to bacterial dissemination *in vivo*, we investigated the ability of *C. rodentium* to attach to the intestinal epithelium. Mice lacking DOCK2 failed to resist early colonization on enterocytes by *C. rodentium* via a yet-undefined mechanism. Although the expression of many genes or proteins encoding mucin, pro-inflammatory cytokines and other anti-microbial peptides were not impaired in *Dock2*^−/−^ mice, it is possible that other anti-microbial factors produced by intestinal epithelial cells or immune cells may account for the differential susceptibility to enterocyte attachment by *C. rodentium* in WT and *Dock2*^−/−^ mice.

Rac1 and Rac2 are essential components mediating the function of DOCK2 34. It is possible that DOCK2 signals via Rac1 and/or Rac2 in the host defense against *C. rodentium* infection. However, unlike mice lacking DOCK2, mice deficient in Rac2 are susceptible to *C. rodentium* infection only after 12 days of infection^35^. Further studies directly comparing *Dock2*^−/−^, *Rac1*^−/−^, and *Rac2*^−/−^ mice will help clarify the signaling components governing DOCK2-mediated defense during *C. rodentium* infection.

The functional role of DOCK2 is likely to extend beyond the gastrointestinal tract. Mouse microglial cells lacking DOCK2 have an impaired ability to produce pro-inflammatory cytokines in response to LPS^36^, indicating a potential immunological role for DOCK2 in the central nervous system. Further studies are required to elucidate the biological functions of DOCK2 in response to different pathogens. In conclusion, our study identified DOCK2 as an important regulator of host defense against *C. rodentium* infection.

## Methods

### Mice

*Dock2*^−/−^ mice have been described previously^14^. C57BL/6 mice were used as WT controls. Mice were housed in a pathogen-free facility. Animal procedures were approved by, and performed in accordance with the relevant guidelines from the St. Jude Children’s Research Hospital Committee on Use and Care of Animals.

### Infection

*Citrobacter rodentium* (ATCC #51459) was grown in pre-warmed LB broth for 9 h at 37 °C with shaking. Mice were fasted for 4 h prior to infection with 1 × 10^10^ CFU per mouse by oral gavage. Spleen, liver, colon, MLN and fecal pellets were harvested as described previously^37^. To determine bacterial counts, serial dilutions of homogenized tissues and fecal pellets were plated on MacConkey agar plates and incubated for 24 h at 37 °C.

### Histology and immunohistochemistry

Colons were fixed in 10% formalin, embedded in paraffin, sectioned and stained with H&E as described previously^38^. *C. rodentium* Tir was immunostained with *C. rodentium*-specific Tir antibody (gift from W. Deng and B.B. Finlay, University of British Columbia, Canada). Macrophages and neutrophils were stained with anti-F4/80 (1:500 dilution, MS48000, Caltag) and anti-neutrophils 7/4 (1:2,500 dilution, RM6500, Caltag) antibodies, respectively. Histological findings, including inflammation, edema, hyperplasia, the extent of colonic damage and crypt length, were evaluated at St. Jude Children’s Research Hospital by a pathologist in a blinded fashion.

### Electronic Microscopy

Intestinal tissue samples were fixed in 2.5% glutaraldehyde and 2% paraformaldehyde in 0.1 M sodium cacodylate buffer (pH 7.4) and post-fixed for 1.5 h in 2% osmium tetroxide in 0.1 M sodium cacodylate buffer supplemented with 0.3% potassium ferrocyanide. After rinsing in the post-fixed buffer, samples were dehydrated through a series of graded ethanol to propylene oxide buffers, and infiltrated and embedded in epoxy resin, followed by polymerization at 70 °C overnight. Semi-thin sections of 0.5 micron thickness were prepared and stained with toluidine blue for light microscope examination. Ultrathin sections of 80 nm thickness were sectioned and imaged using a FEI Tecnai F 20 TEM FEG Electron Microscope (FEI, Hillsboro) equipped with an ATM XR41 camera.

### Cytokine analysis

Colon tissues were homogenized in RIPA buffer supplemented with protease and phosphatase inhibitors (Roche). Levels of cytokines and chemokines in colon homogenates and sera were determined by multiplex ELISA according to the manufacturer’s instructions (Millipore).

### Quantitative RT-PCR

RNA was isolated using Trizol, followed by conversion to cDNA as described previously^38^. Real-time quantitative PCR was performed on an ABI 7500 real-time PCR instrument with 2 ×  SYBR Green kit (Applied Biosystems) and the appropriate primers (sequences are found in Supplementary Table S1).

### Statistical analysis

GraphPad Prism 6.0 software was used for data analysis. Data are shown as mean ± SEM. Statistical significance was determined by *t* tests (two-tailed) for two groups. Body weight change was compared using two-way ANOVA. Survival curves were compared using the log-rank test. P < 0.05 was considered statistically significant.

## Additional Information

**How to cite this article**: Liu, Z. *et al*. DOCK2 confers immunity and intestinal colonization resistance to *Citrobacter rodentium* infection. *Sci. Rep.* **6**, 27814; doi: 10.1038/srep27814 (2016).

## Supplementary Material

Supplementary Information
