# Supplementary material for: DOCK2 confers immunity and intestinal colonization resistance to Citrobacter rodentium infection
Source: Sci Rep. 2016 Jun 13;6:27814. doi: 10.1038/srep27814 (PMC4904218; doi:10.1038/srep27814)
Supplement: Supplementary Information [file srep27814-s1.pdf]

# SUPPLEMENTARY INFORMATION

# DOCK2 confers immunity and intestinal colonization resistance to *Citrobacter rodentium* infection

Zhiping Liu[1], Si Ming Man[1], Qifan Zhu[1,2], Peter Vogel[3], Sharon Frase[4], Yoshinori Fukui[5], and Thirumala-Devi Kanneganti[1,*]

[1] Department of Immunology, St. Jude Children's Research Hospital, Memphis, TN, 38105, USA;

[2] Integrated Biomedical Sciences Program, University of Tennessee Health Science Center, Memphis, Tennessee 38163, USA;

[3] Animal Resources Center and the Veterinary Pathology Core, St. Jude Children's Research Hospital, Memphis, TN, 38105, USA;

[4] Cell and Tissue Imaging Center, St. Jude Children's Research Hospital, Memphis, TN, 38105, USA;

[5] Division of Immunogenetics, Department of Immunobiology and Neuroscience, Medical Institute of Bioregulation, Kyushu University, Fukuoka 812-8582, Japan.

*Correspondence: Thirumala-Devi Kanneganti, Department of Immunology, MS 351, St Jude Children's Research Hospital, 262 Danny Thomas Place, Memphis, TN 38105-3678

Tel: (901) 595-3634; Fax. (901) 595-5766.

E-mail: Thirumala-Devi.Kanneganti@StJude.org.

**Supplementary figures legends**

**Supplementary Figure S1. *DOCK2*$^{-/-}$ mice do not have an impaired ability to produce cytokines during *C. rodentium* infection**. (A) Levels of IL-17, IFN-γ and TNF-α proteins in the colon 14 days post-infection with *C. rodentium*. (B and C) Real-time qPCR analysis of the expression of genes encoding IL-23p19 (B) and IL-22 (C) in colon tissues of WT and *Dock2*$^{-/-}$ mice infected with *C. rodentium*. Data are representative of two independent experiments (mean and SEM). Two-tailed *t*-test. *, P < 0.05; NS, not statistically significant.

**Supplementary Figure S2. *DOCK2*$^{-/-}$ mice have similar expression of the gene encoding mucin**. Real-time qPCR analysis of the expression of genes encoding MUC1, MUC, MUC3 and MUC4 in the colon of WT and *DOCK2*$^{-/-}$ mice 4 days post-infection with *C. rodentium*.. Data are representative of two independent experiments (mean and SEM). Two-tailed *t*-test. NS, not statistically significant.

**Supplementary Table S1. Real time qPCR primer sequence.**

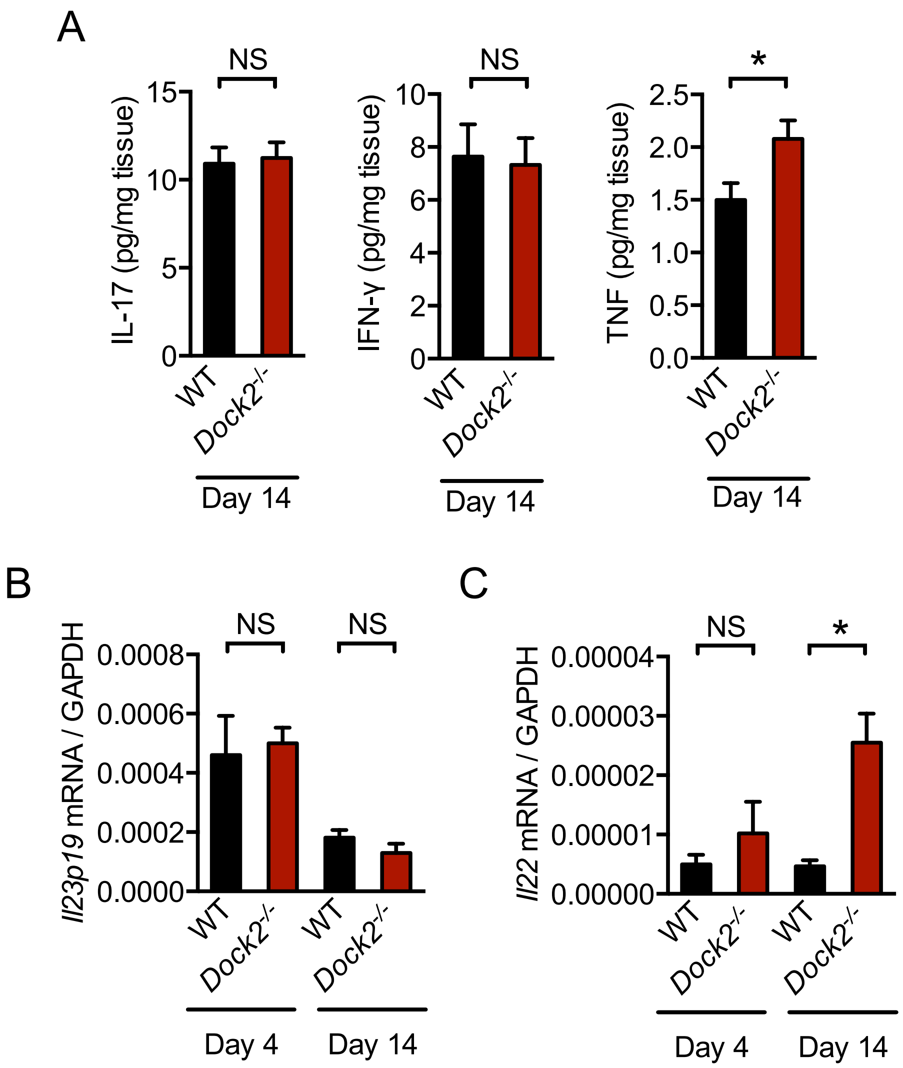


**Supplementary Figure S1**

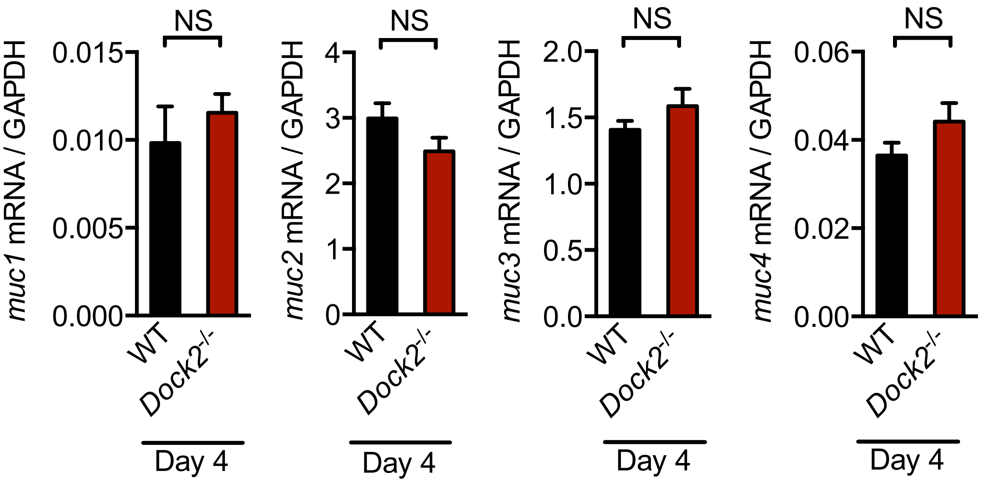


**Supplementary Figure S2**
